# Anti-Inflammatory Activity and ROS-Inhibitory Effect of Gomphrenin-Enriched Preparations

**DOI:** 10.3390/nu18203410

**Published:** 2026-10-17

**Authors:** Monika E. Czerwińska, Katarzyna Sutor-Świeży, Daria Berezovska, Mateusz Knap, Sławomir Wybraniec, Adam Matkowski, Sylwia Zielińska

**Affiliations:** 1 Department of Biochemistry and Pharmacogenomics, Faculty of Pharmacy, Medical University of Warsaw, 02-097 Warsaw, Poland; monika.czerwinska@wum.edu.pl (M.E.C.);; 2 Centre for Preclinical Research, Medical University of Warsaw, 02-097 Warsaw, Poland; 3 Cracow University of Technology, Faculty of Chemical Engineering and Technology, Department C-1, 31-155 Krakow, Polandmateusz.knap@doktorant.pk.edu.pl (M.K.); slawomir.wybraniec@pk.edu.pl (S.W.); 4 Cracow University of Technology, CUT Doctoral School, Faculty of Chemical Engineering and Technology, Department C-1, 31-155 Cracow, Poland; 5 Division of Pharmaceutical Biology and Botany, Department of Pharmaceutical Biology and Biotechnology, Faculty of Pharmacy, Wroclaw Medical University, 50-556 Wroclaw, Poland; 6 Division of Pharmaceutical Biotechnology, Department of Pharmaceutical Biology and Biotechnology, Faculty of Pharmacy, Wroclaw Medical University, 50-556 Wroclaw, Poland

**Keywords:** gomphrenins, *Basella*, *Gomphrena*, anti-inflammatory effect, ROS

## Abstract

**Background/Objectives:** Betanidin glycosides (betacyanins) are plant dietary pigments with reported significant benefits on human health. Betacyanin research has largely focused on betanin common in some plant foods like beets, amaranth, quinoa, cacti fruits, whereas the biological properties of structurally diverse gomphrenin derivatives remain insufficiently characterized. Gomphrenin-type betacyanin dietary source is predominantly Malabar spinach (*Basella alba* L.) but its health-promoting and disease-preventing properties remain understudied. This study investigated whether differences in the composition of gomphrenin-rich preparations influence their effects on inflammatory and oxidative cellular responses. **Methods:** Betacyanin-rich preparations were obtained from *Basella alba* f. *rubra* fruits and *Gomphrena globosa* L. flowers using chromatographic purification. Their betacyanin profiles were characterized by LC-DAD-ESI-MS and total betacyanin contents were determined spectrophotometrically. The biological effects of crude and purified preparations (1–100 μg/mL) were evaluated in Caco-2 intestinal epithelial cells and human polymorphonuclear neutrophils (PMN). IL-8 secretion in Caco-2 cells, TNF-*α*, IL-8, and IL-1*β* secretion in LPS-stimulated PMN, ROS generation in f-MLP-stimulated PMN, and cell viability were assessed. **Results:** Purification resulted in preparations with markedly different betacyanin profiles. The gomphrenin-enriched BE_2_ contained 312 ± 83 mg/g total betacyanins, whereas the acylated-betacyanin-enriched BE_3_ contained 188 ± 12 mg/g and was dominated by globosin and other hydroxycinnamoyl derivatives. Despite its lower total betacyanin content, BE_3_ displayed stronger inhibition of TNF-*α*, IL-8, and IL-1*β* secretion than BE_2_; at 100 μg/mL, these cytokine levels were 39.17%, 44.61%, and 58.93% of the LPS control, respectively. All preparations reduced IL-8 secretion in Caco-2 cells by more than 50% at the highest concentration and the *Basella* preparations effectively suppressed neutrophil ROS generation. No significant cytotoxicity was observed. **Conclusions:** The biological activity of betalain-rich preparations was not determined by total betacyanin content alone but was associated with their qualitative pigment composition and broader chemical matrix. The stronger anti-inflammatory activity of the acylated-betacyanin-enriched preparation highlights hydroxycinnamoylated gomphrenins as candidates for further investigation, while the complex nature of the preparations warrants further studies to determine the contribution of individual pigments to the observed biological effects.

## 1. Introduction

The growing interest in plant-derived bioactive compounds has shifted from identifying molecules with antioxidant capacity toward elucidating their specific mechanisms of action in chronic inflammatory processes [1]. Inflammation is an essential component of host defense and tissue repair; however, persistent or dysregulated inflammatory responses can contribute to tissue damage and disease progression. In the intestine, inflammatory processes are particularly complex because epithelial cells and immune cells continuously interact with dietary components, microbial products, and endogenous danger signals. Excessive production of inflammatory mediators, increased oxidative stress, and impairment of epithelial barrier function are not independent events but form a closely interconnected network that can sustain and amplify intestinal inflammation [2,3,4]. Consequently, evaluation of potential anti-inflammatory agents requires consideration of multiple cellular processes rather than reliance on a single endpoint. While numerous phytochemicals have demonstrated anti-inflammatory potential in vitro and in vivo, it has become increasingly evident that structurally related metabolites may differ substantially in their biological activity [5,6]. Among naturally occurring nitrogen-containing pigments, betalains have emerged as promising modulators of inflammation. However, current knowledge is strongly biased toward betanin from red beet (*Beta vulgaris*), whereas other naturally occurring betalain structures remain largely unexplored [7,8]. This imbalance limits our understanding of how structural modifications within the betalain scaffold influence biological activity and whether individual betalain subclasses exhibit distinct anti-inflammatory properties. Particularly little is known about gomphrenins and their naturally occurring derivatives, despite their considerable structural diversity resulting from variations in glycosylation, acylation, and decarboxylation patterns [9,10]. These structural modifications may affect not only pigment stability and bioavailability but also interactions with molecular targets involved in inflammatory signaling. Betalains are a unique class of water-soluble nitrogen-containing pigments naturally occurring in plants of the order Caryophyllales [11,12]. Beyond their well-established role as natural colorants, these compounds have attracted considerable scientific interest due to their diverse biological activities, including antioxidant, anti-inflammatory, anticancer, and cardioprotective effects [13]. Among these properties, their anti-inflammatory potential has emerged as a particularly promising area of research, given the central role of chronic inflammation in the development of numerous non-communicable diseases. Current evidence suggests that betalains modulate inflammatory responses through multiple molecular mechanisms, including regulating oxidative stress, inhibiting pro-inflammatory signaling pathways such as nuclear factor κ-light-chain-enhancer of activated B cells (NF-κB) and mitogen-activated protein kinases (MAPK), and suppressing the production of inflammatory mediators and cytokines [14,15]. While most available studies have focused on betanin and related pigments from red beet, far less attention has been paid to betalains in other plant species. Gomphrenins, a distinctive group of betacyanins predominantly found in species of the genus *Gomphrena* and several other representatives of the family Amaranthaceae, represent a structurally diverse subclass of betalains with potentially unique biological properties [16,17]. Despite increasing interest in their chemistry, stability, and biosynthesis, knowledge regarding their anti-inflammatory activity remains limited. Therefore, a comprehensive evaluation of the anti-inflammatory potential of gomphrenin derivatives is warranted to better understand their biological functions and assess their potential application as naturally derived bioactive candidates for future functional food and nutraceutical applications.

An important question is whether the biological effects of betalain-rich preparations can be predicted from the properties of individual pigments, or whether they depend on the composition of the preparation as a whole. Preparations differing in their gomphrenin composition may therefore exhibit distinct effects on inflammatory responses, and their biological activity cannot necessarily be extrapolated from the effects previously reported for betanin. To address this issue, the present study investigated preparations obtained from *Basella alba* f. *rubra* and *Gomphrena globosa*, including crude and purified fractions, with particular focus on their effects on cellular responses associated with inflammation and oxidative stress. The comparison of crude and purified *B. alba* preparations further provided an opportunity to examine how changes in chemical composition may influence biological activity and whether enrichment of specific constituents, removal of potentially antagonistic compounds, or altered interactions among co-occurring metabolites contribute to differences in cellular responses.

## 2. Materials and Methods

### 2.1. Plant Material

*Basella alba* L. forma *rubra* plants were cultivated in the greenhouse of the Agricultural University of Krakow (Faculty of Biotechnology and Horticulture) from seeds obtained from the Botanical Garden of the Jagiellonian University Institute of Botany (Cracow, Poland). The seeds were sown in a 3:1 ratio of soil and coconut pith mass and watered daily. The seedlings were transplanted to fertile soil with plenty of organic matter and a pH of 6.5–6.8. The plants were designed to support the fast climbing of the vines which were trellised so that they reached up to 3 m in length at controlled humidity and temperature, ensuring continuous flowering and fruiting as described in our previous article [18]. After harvesting, the fruits were stored at −18 °C until further use.

Dried flowers of purple variety *Gomphrena globosa* L. were purchased from a local market and authenticated using morphoanatomical comparison to living plants from the certified collection of Botanical Garden of Medicinal Plants at the Wroclaw Medical University (Republic of Poland Ministry of Environment license No. DOPogiz-4210-26-6024-/05/kl).

Dried voucher specimens are preserved in the Botanical Garden of Medicinal Pants herbarium (Basella/alba/rubra/PK/2026/1A and Gomphrena/globosa/purple/BGMP/2026/1).

### 2.2. Sample Preparation

The fruits were extracted with demineralized water for 1 h at room temperature (0.6 L per 1 kg of raw material). During extraction, the fruits were gently crushed (without damaging the seeds) to enhance the extraction efficiency. Then the crushed fruits were left in the extraction solution without further agitation. The liquid extract was separated from the plant material by centrifugation at 1300 rpm, with the plant material contained in a nylon filter bag. The extraction was repeated three times. The combined extracts were frozen and freeze-dried, yielding *B. alba* fruit extract (BE) which was stored at −18 °C prior to further studies.

For the preparation of betacyanin-enriched *B. alba* fruit extract (BE_1_), the raw material was extracted as described above, except that EDTA was added during extraction (0.4 mL of saturated EDTA solution per 1 L of water), after which the extract was stored as described above, then filtered under reduced pressure through a layer of silica gel with a particle size of 60–200 µm (VWR, Radnor, PA, USA), and diluted with water before further use. The prepared extract was purified using a weak anion-exchange resin (Sepra™ ZT WAX, Phenomenex, Torrance, CA, USA), which had been acetone-activated and water-conditioned. After pigment adsorption, the resin was washed with demineralized water. The pigments were eluted with an aqueous solution containing 50% (*v*/*v*) acetone and 5% (*v*/*v*) formic acid, followed by 50% (*v*/*v*) acetone and 10% (*v*/*v*) formic acid. The eluate was concentrated under reduced pressure at 22 °C using a rotary evaporator, diluted with demineralized water and applied to a hydrophobic ODS C18 sorbent (Chromabond, Macherey & Nagel, Düren, Germany), which had been acetone-activated and water-conditioned. After washing with demineralized water, the pigments were eluted with an aqueous solution containing 50% (*v*/*v*) acetone and 5% (*v*/*v*) acetic acid, followed by 50% (*v*/*v*) acetone and 10% (*v*/*v*) acetic acid. The eluate was concentrated, freeze-dried, and stored at −18 °C.

The betanidin 6-*O*-*β*-glucoside (gomphrenin)-enriched fraction (BE_2_) and the 6′-*O*-*trans*-4-coumaroyl-gomphrenin (globosin)-enriched fraction (BE_3_) were obtained as follows. The fruit extract prepared as described for BE_1_ was acidified with hydrochloric acid to 0.04 M and loaded onto a strong cation-exchange resin with sulfonic acid functional groups. The column was washed sequentially with 0.1 M hydrochloric acid, 5% (*v*/*v*) formic acid and demineralized water. The pigments were eluted with 50% (*v*/*v*) aqueous acetone, with EDTA added to the eluate during elution (0.1 mL of saturated EDTA solution per 1 L of eluate). The eluate was concentrated and purified using a hydrophobic ODS C18 sorbent as described for the BE_1_ sample, then separated using preparative high-performance liquid chromatography (HPLC) (Shimadzu Corp., Kyoto, Japan) on a Varian Dynamax Microsorb C18 column (41 × 250 mm, particle size 8 µm; Varian, Palo Alto, CA, USA). Mobile phase A consisted of 1% (*v*/*v*) aqueous formic acid and phase B of acetone. The gradient of phase B was: 6% (*v*/*v*) at 0–15 min, 8% (*v*/*v*) at 15–25 min, 10% (*v*/*v*) at 25–35 min, 15% (*v*/*v*) at 35–45 min, 20% (*v*/*v*) at 45–55 min, 30% (*v*/*v*) at 55–63 min, and 70% (*v*/*v*) at 63–70 min, at a flow rate of 40 mL/min. Fractions containing the target pigments were selected based on monitoring by a liquid chromatography coupled with diode array detection and electrospray ionization mass spectrometry (LC-DAD-ESI-MS) system, concentrated, freeze-dried, and stored at −18 °C.

The enriched extract from dried purple flowers of *G. globosa* L. (GE) was obtained by extracting plant material with 1% (*v*/*v*) aqueous formic acid solution (30 L per 1 kg) supplemented with saturated EDTA solution (0.2 mL per 10 L). The obtained extract was subsequently filtered through silica gel and purified using an anion-exchange resin and a hydrophobic ODS C18 sorbent, as described for the BE_1_ sample, except that strong anion-exchange resin (Sepra™ ZT-SAX, Phenomenex, Torrance, CA, USA) was used instead of a weak anion-exchange resin, as previously reported [9]. The eluate was concentrated, freeze-dried, and stored at −18 °C.

### 2.3. Chromatographic Analysis Using Liquid Chromatography–Diode Array Detection–Electrospray Ionization Mass Spectrometry (LC-DAD-ESI-MS)

For the analysis of extracts and fractions collected during purification, a low-resolution LC-MS-8030 mass spectrometric system (Shimadzu, Kyoto, Japan) coupled to LC-20ADXR HPLC pumps, an injector model SIL-20ACXR, and a PDA detector (photodiode array) model SPD-M20A, all controlled with LabSolutions software, version 5.60 SP1 (Shimadzu), was applied [9,18].

The samples were eluted through a 150 mm × 4.6 mm i.d., 5.0 μm, Kinetex C18 chromatographic column preceded by a guard column of the same material (Phenomenex, Torrance, CA, USA). The injection volume was 7 μL, and the flow rate was 0.5 mL/min. The column was thermostated at 40 °C. The separation of the analytes was performed with binary gradient elution. The mobile phases were A, 1% (*v*/*v*) formic acid in water and B, methanol. The gradient profile was (t (min), % B), (0, 10), (12, 55), (15, 85). The full-range PDA signal was recorded, and individual chromatograms were displayed at 538, 505, 490, and 440 nm. Positive ion electrospray mass spectra were recorded using the LC-MS system. The ionization electrospray source operated in positive mode (ESI+), with an electrospray voltage of 4.5 kV and a capillary temperature of 250 °C. N_2_ gas was used for the spray. Argon was used as the collision gas for the collision-induced dissociation (CID) experiments. The relative collision energies for MS/MS analyses were set at −35 V on an arbitrary scale. The system recorded total ion chromatograms, mass spectra, and ion chromatograms in the selected ion monitoring mode (SIM). The normalized composition of detected betacyanins was calculated from the peak areas of the corresponding SIM chromatographic signals. The sum of the peak areas of the detected signals assigned to identified betacyanins was set to 100%.

### 2.4. Determination of Total Betacyanin Content

To determine the total betacyanin content, the samples were analyzed spectrophotometrically using an Infinite 200 PRO microplate reader (Tecan, Männedorf, Switzerland).

The total betacyanin concentration was calculated according to the Beer-Lambert law:

*A* = *C*
**∙**
*ε*
**∙**
*l*,

where *A* is the mean absorbance, *C* is the molar concentration of the analyte in the solution (mol dm^−3^), ***l*** is the optical path length (cm), and ***ε*** is the molar extinction coefficient (dm^3^ mol^−1^ cm^−1^).

A molar extinction coefficient (*ε*) of 65,000 dm^3^ mol^−1^ cm^−1^ and an optical path length (*l*) of 0.53 cm were used for the calculations. The absorbance values used in the calculations corresponded to the absorption maxima recorded within the wavelength range of 500–570 nm. The total betacyanin content was calculated using the molecular weight of the dominant pigment: 550 g mol^−1^ for BE, BE_1_ and BE_2_; 696 g mol^−1^ for BE_3_; 726 g mol^−1^ for GE.

### 2.5. Biological Studies

#### 2.5.1. Reagents

Dimethyl sulfoxide (DMSO, 113635509) was purchased from Chempur (Piekary Śląskie, Poland). Citric acid, sodium citrate tribasic dihydrate, and glucose for acid citrate dextrose solution (ACD) were purchased from Chempur (Piekary Śląskie, Poland). Dextran from *Leuconostoc mesenteroides*, propidium iodide, dexamethasone, luminol, formyl-met-leu-phenylalanine (f-MLP), sodium butyrate, and Triton X-100 were purchased from Sigma-Aldrich (Saint Louis, MO, USA). RPMI 1640 medium, amphotericin B, and 3-(4,5-dimethylthiazol-2-yl)-2,5-diphenyltetrazolium (MTT) were purchased from Sigma-Aldrich (Saint Louis, MO, USA). Pancoll Human was purchased from PAN-Biotech (Aidenbach, Germany). Phosphate-buffered saline (PBS) and Dulbecco’s modified Eagle medium (DMEM) enriched with 3.7 g/L NaHCO_3_ and 4.5 g/L glucose, with stable L-glutamine, low endotoxin, fetal bovine serum (FBS) as well as penicillin-streptomycin were purchased from Biowest (Nauillé, France). Hanks’ balanced salt solution (HBSS) and TrypLE™ Express Enzyme were purchased from Gibco Thermo Fisher (Waltham, MA, USA). Recombinant human IL-1*β* protein, LPS from *Escherichia coli* (0111:B4), and formic acid were obtained from Merck (Darmstadt, Germany). Recombinant tumor necrosis factor-α (TNF-α) as well as recombinant inteferon-*γ* were purchased from Pepro-Tech (Cranbury, NJ, USA). Sets of immunosorbent assays for human IL-8, TNF-*α*, and IL-1*β* were purchased from BD Biosciences (Erembodegem, Belgium). Pierce BCA Protein Assay Kit (23225) was obtained from Thermo Fisher Scientific (Rockford, IL, USA).

Human adenocarcinoma cell line Caco-2 (ACC 169), purchased from Leibniz Institute DSMZ-German Collection of Microorganisms and Cell Cultures GmbH (Braunschweig, Germany), was used for cytotoxicity and IL-8 secretion assays. Neutrophils-type PMN (polymorphonuclear cells) were obtained from buffy coats procured at the Warsaw Blood Donation Centre (Warsaw, Poland).

#### 2.5.2. Cytotoxicity and IL-8 Assay in Caco-2 Cell Line

Caco-2 cells were cultured in 75 mL flasks at a seeding density of 10^5^ cells/mL in DMEM supplemented with 20% FBS, 2 mM L-alanyl-L-glutamine, 100 IU/mL penicillin, 100 µg/mL streptomycin, and 2.5 ng/mL amphotericin B. Cultures were maintained at 37 °C in a humidified atmosphere with 5% CO_2_. Cells in passages ranging from 11 to 28 were used for all experiments.

Cytotoxicity was assessed via an MTT assay. Cells treated with extracts (1–100 µg/mL) for 48 h were washed, incubated with MTT (0.5 mg/mL), and dissolved in DMSO. Absorbance was measured at 560 nm and 620 nm using a microplate reader (Biotek Epoch, Winooski,, VT, USA). Triton X-100 (10%, *v*/*v*) served as the positive control.

For experiments, cells were seeded in 24-well plates (2 × 10^5^ cells/well) in 20% FBS medium. After 24 h, the medium was replaced with DMEM containing 5% FBS, 2 mM L-alanyl-L-glutamine, 100 IU/mL penicillin, 100 µg/mL streptomycin, 2.5 ng/mL amphotericin B, and 5 mM sodium butyrate to induce differentiation for 72 h. Differentiated enterocyte-like monolayer cells were treated overnight with extracts (1, 10, 50, and 100 µg/mL) before stimulation with IL-1*β* (25 ng/mL), TNF-*α* (10 ng/mL), IFN-*γ* (10 ng/mL), and LPS (100 ng/mL) to induce IL-8 secretion, as it was previously described. After 24 h, supernatants were collected, and IL-8 levels were quantified using ELISA and normalized to protein content with the Pierce™ BCA Assay Kit. Dexamethasone (5–100 µM) served as a positive control [19].

#### 2.5.3. The Cytotoxicity, ROS, and Cytokine Assays in PMN

Neutrophils were isolated from buffy coats obtained from healthy volunteers (<35 years old) at the Warsaw Blood Donation Centre, using anticoagulant citrate dextrose solution (ACD). Isolation involved dextran sedimentation, density gradient centrifugation (Pancoll Human, 1.077 g/mL), and erythrocyte removal via water-induced hemolysis. The cells were suspended in RPMI 1640 or HBSS and stored at 4 °C until use.

For ROS production assays, neutrophils (3.5 × 10^5^ cells/well) in HBSS were seeded in 96-well plates, while cytokine secretion assays used 2 × 10^6^ cells/well in RPMI 1640 with 10% FBS, 2 mM L-glutamine, and 10 mM HEPES. Cell cytotoxicity was assessed via flow cytometry, with cells treated with extracts (1, 10, 50, and 100 µg/mL) for 24 h. Following treatment, cells were stained with propidium iodide (PI) and analyzed on a BD FACSVerse (BD Biosciences, San Jose, CA, USA). Cytotoxicity was expressed as a percentage of PI(+) cells, with Triton X-100 as the positive control.

ROS production by f-MLP-stimulated neutrophils was measured using chemiluminescence. Cells were treated with extracts (1–100 µg/mL), luminol (2.5 mM), and f-MLP (1.5 µg/mL). Luminescence was recorded over 40 min in a microplate reader (SYNERGY 4, Biotek, Winooski, VT, USA), and inhibition percentages were calculated against non-stimulated controls (n.st. control) [19]. Ascorbic acid (ASC) was used as a positive control at concentrations 1 (5.7 µM), 25 (142 µM), and 50 (268 µM) µg/mL.

Cytokine secretion (IL-8, TNF-*α*, IL-1*β*) was measured by ELISA after LPS stimulation (100 ng/mL). Neutrophils were pre-treated with extracts (1–100 µg/mL) for 30 min and cultured for 24 h. Results were expressed relative to LPS-stimulated controls, with dexamethasone (Dex) as the positive control [19].

### 2.6. Statistical Analysis

Data on the normalized composition of detected betacyanins in the studied preparations determined by LC-DAD-ESI-MS analysis, and total betacyanin contents determined spectrophotometrically, are presented as mean ± standard deviation (SD) of three independent measurements.

Biological data are presented as mean ± standard error of the mean (S.E.M). Each sample was analyzed in triplicate across three independent experiments. Statistical differences between means were assessed by verifying variance homogeneity and normality of distribution, followed by one-way ANOVA. When necessary, non-parametric tests such as Kruskal–Wallis or U-Mann–Whitney were applied. Statistical significance was set at *p* < 0.05. Data analysis was performed using Statistica 13 software (TIBCO Software Inc., Palo Alto, CA, USA). Graphical abstract was created using BioRender (Toronto, ON, Canada).

## 3. Results

### 3.1. Betacyanin Composition of the Purified Preparations

The LC-DAD-ESI-MS analysis demonstrated pronounced differences in both the qualitative and quantitative composition of betacyanins among the obtained preparations (Table 1 and Table 2), reflecting the efficiency of the applied purification procedures.

Considerable differences were observed in the total betacyanin content of the analyzed samples (Table 2). The total betacyanin content in freeze-dried fruit extract BE was 52 ± 4 mg g^−1^.

Purification markedly increased the concentration of betacyanins in the preparations. The total betacyanin content increased to 235 ± 57 mg g^−1^ in BE_1_, 312 ± 83 mg g^−1^ in the gomphrenin-enriched fraction BE_2_ and 188 ± 12 mg g^−1^ in the globosin-enriched fraction BE_3_ (Table 2). The highest betacyanin concentration observed in BE_2_ indicates the efficiency of the preparative HPLC procedure for isolating the major non-acylated betacyanin, gomphrenin and its isoform. In contrast, BE_3_ contained a lower total pigment concentration because this fraction was enriched in naturally less abundant acylated derivatives, particularly globosin and other hydroxycinnamoyl conjugates.

A markedly lower total betacyanin content was determined for the purified *G. globosa* extract (GE), amounting to 12 ± 1 mg g^−1^ (Table 2). The betacyanin profile was characterized by a high proportion of feruloyl-, coumaroyl- and sinapoyl-substituted betacyanins, which is consistent with the characteristic pigment profile previously reported for *G. globosa* [20].

**Figure 1:**
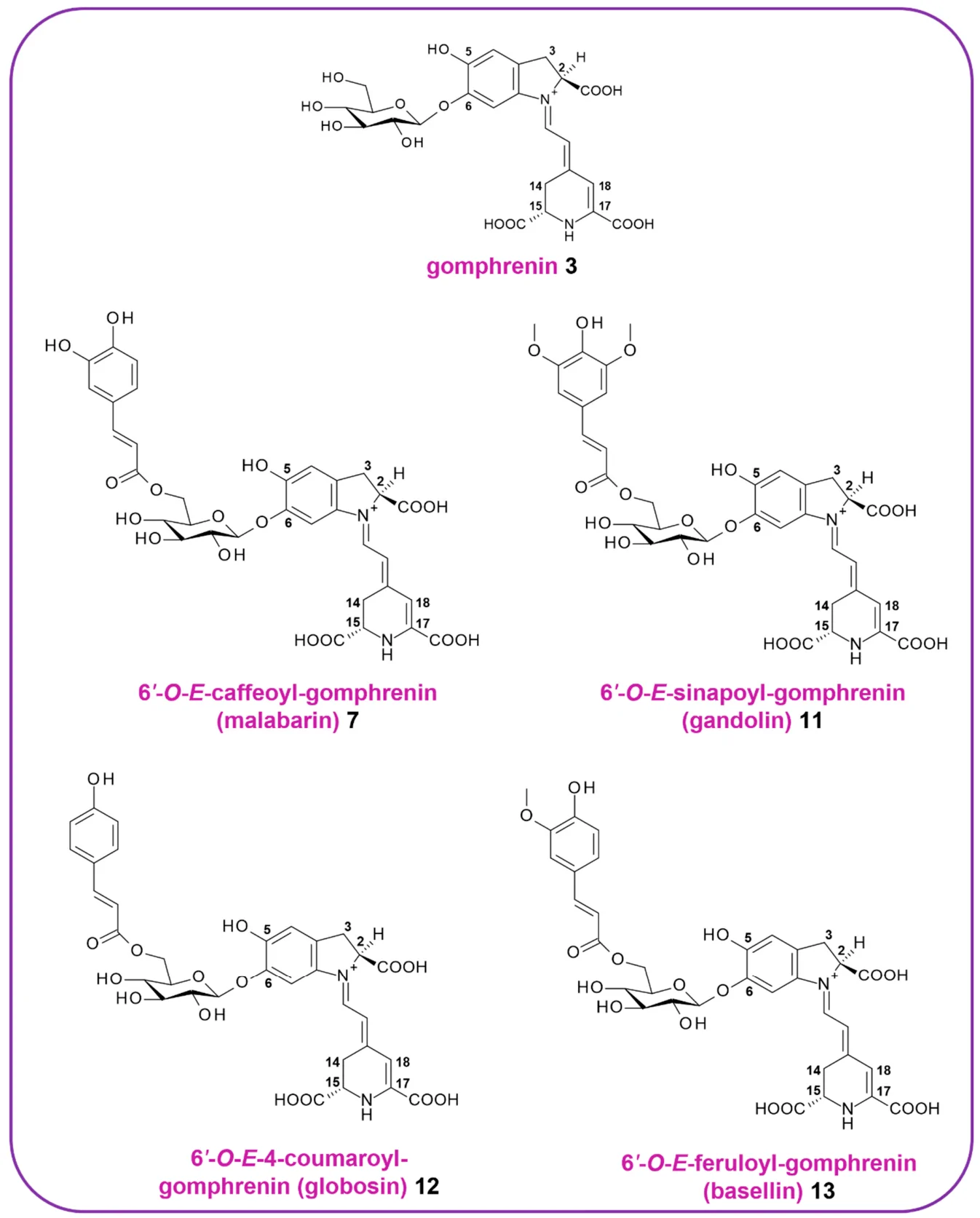
Chemical structures of selected betacyanins. Structure numbers correspond to the compounds listed in Table 1.

**Figure 2:**
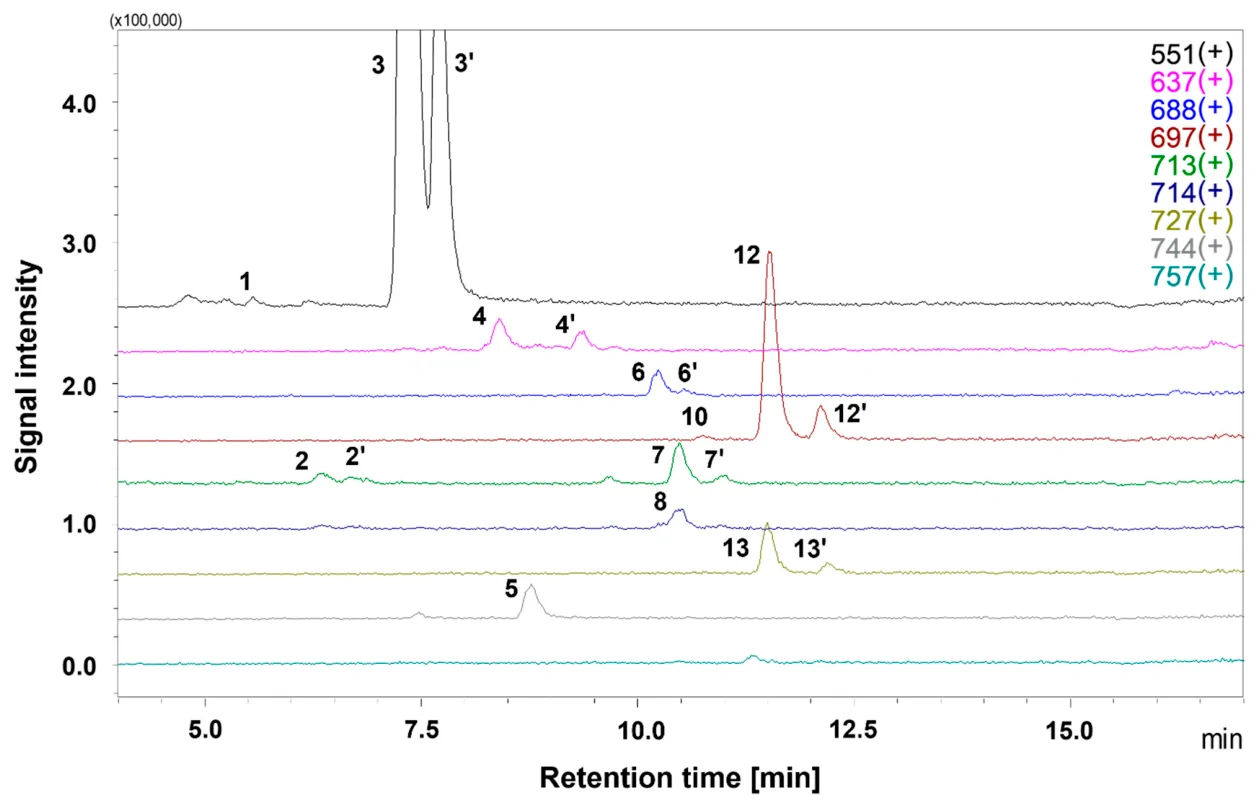
Chromatogram of selected ions monitored in the LC-MS system for BE_1_ preparation. Peak numbers correspond to the compounds listed in Table 1.

Purification of the crude extract on a weak anion-exchange resin followed by ODS C18 chromatography yielded sample BE_1_, which exhibited the highest structural diversity of betacyanins, including non-acylated, malonylated and hydroxycinnamoyl derivatives of gomphrenin and isogomphrenin (Figure 1 and Figure 2). Nevertheless, the betacyanin profile was strongly dominated by gomphrenin (betanidin 6-*O*-*β*-glucoside) **3**, representing 69.7 ± 1.1%, whereas isogomphrenin **3′** accounted for 13.1 ± 1.5% (Table 2).

**Figure 3:**
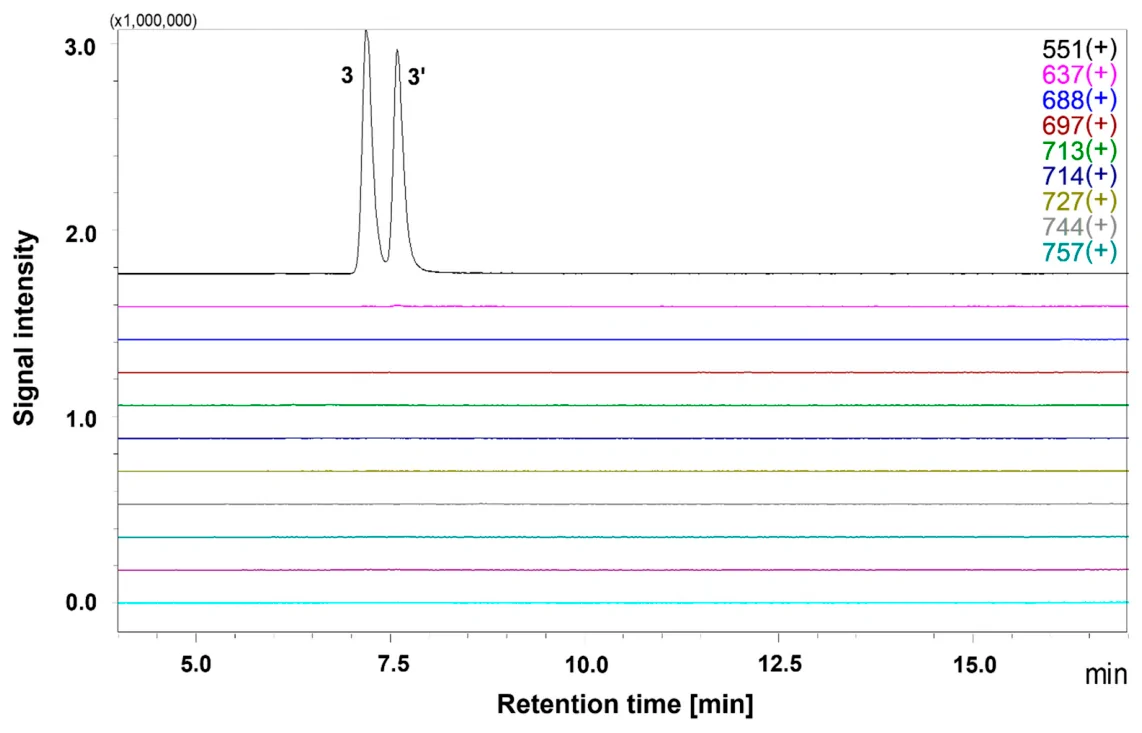
Chromatogram of selected ions monitored in the LC-MS system for gomphrenin-enriched preparation (**BE_2_**). Peak numbers correspond to the compounds listed in Table 1.

The purification strategy employing cation-exchange chromatography followed by preparative HPLC enabled selective enrichment of individual pigments in the betacyanin profile. Sample **BE_2_** represented a gomphrenin-enriched fraction, in which gomphrenin **3** constituted 51.9 ± 4.8% of the detected betacyanins and isogomphrenin **3′** 47.9 ± 4.8%, where malonylated derivatives **4** were detected at low levels (Figure 3). Consequently, more than 99% of the betacyanin composition consisted of the two glucosylated betanidin isomers (Table 2).

**Figure 4:**
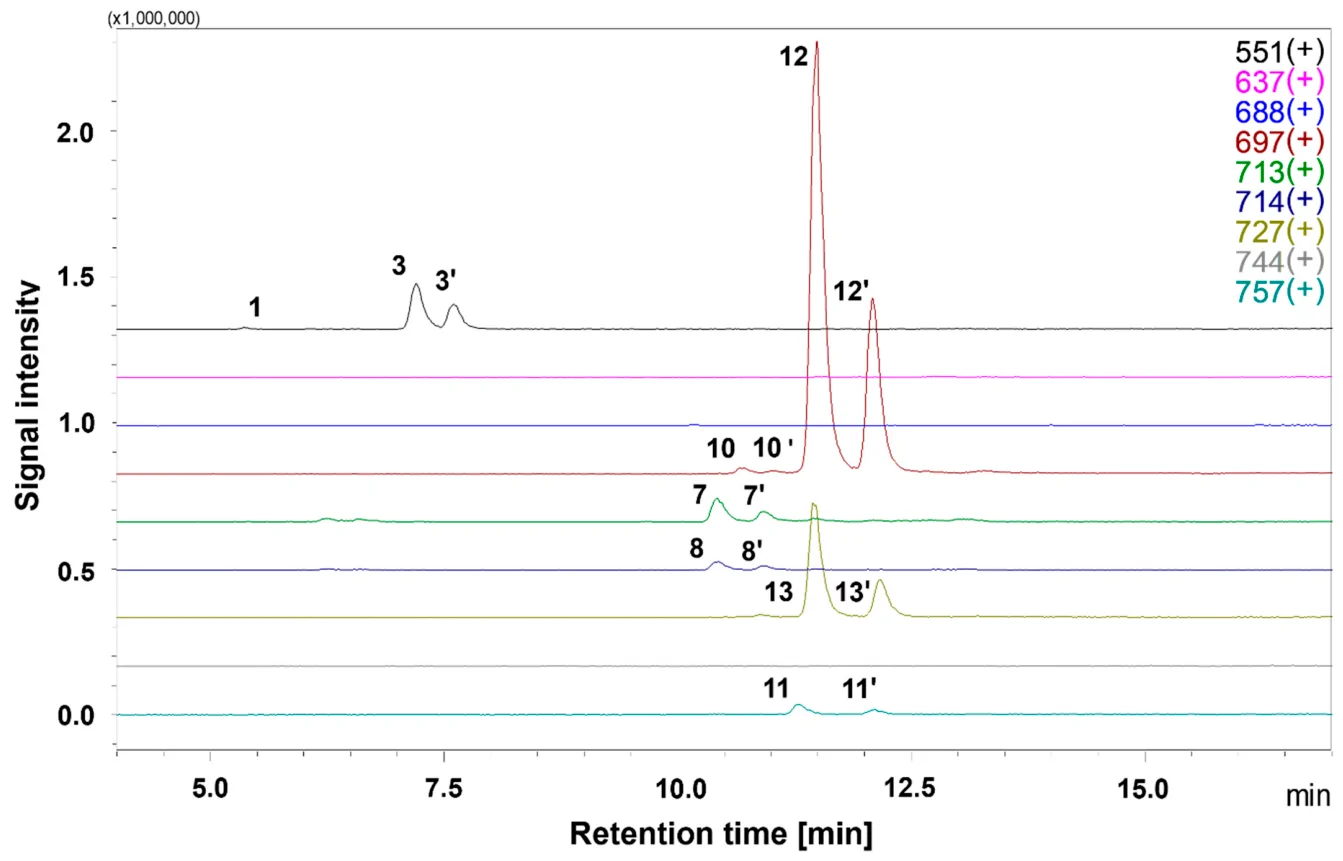
Chromatogram of selected ions monitored in the LC-MS system for acylated pigment-enriched preparation (BE_3_). Peak numbers correspond to the compounds listed in Table 1.

In contrast, sample BE_3_ was enriched in acylated betacyanins (Figure 4). The predominant compound in the betacyanin profile was globosin (6′-*O*-*E*-4-coumaroyl-gomphrenin) **12**, accounting for 48.3 ± 0.5%, followed by 6′-*O*-*E*-feruloyl-gomphrenin (basellin) **13** (12.8 ± 0.1%) and its isoform. Only a residual amount of gomphrenin **3** was detected in the prepared fraction (4.6 ± 0.3%) (Table 2). The considerable proportion of hydroxycinnamoyl derivatives demonstrates that preparative HPLC effectively separated the less hydrophilic acylated pigments from the parent glucosides.

**Figure 5:**
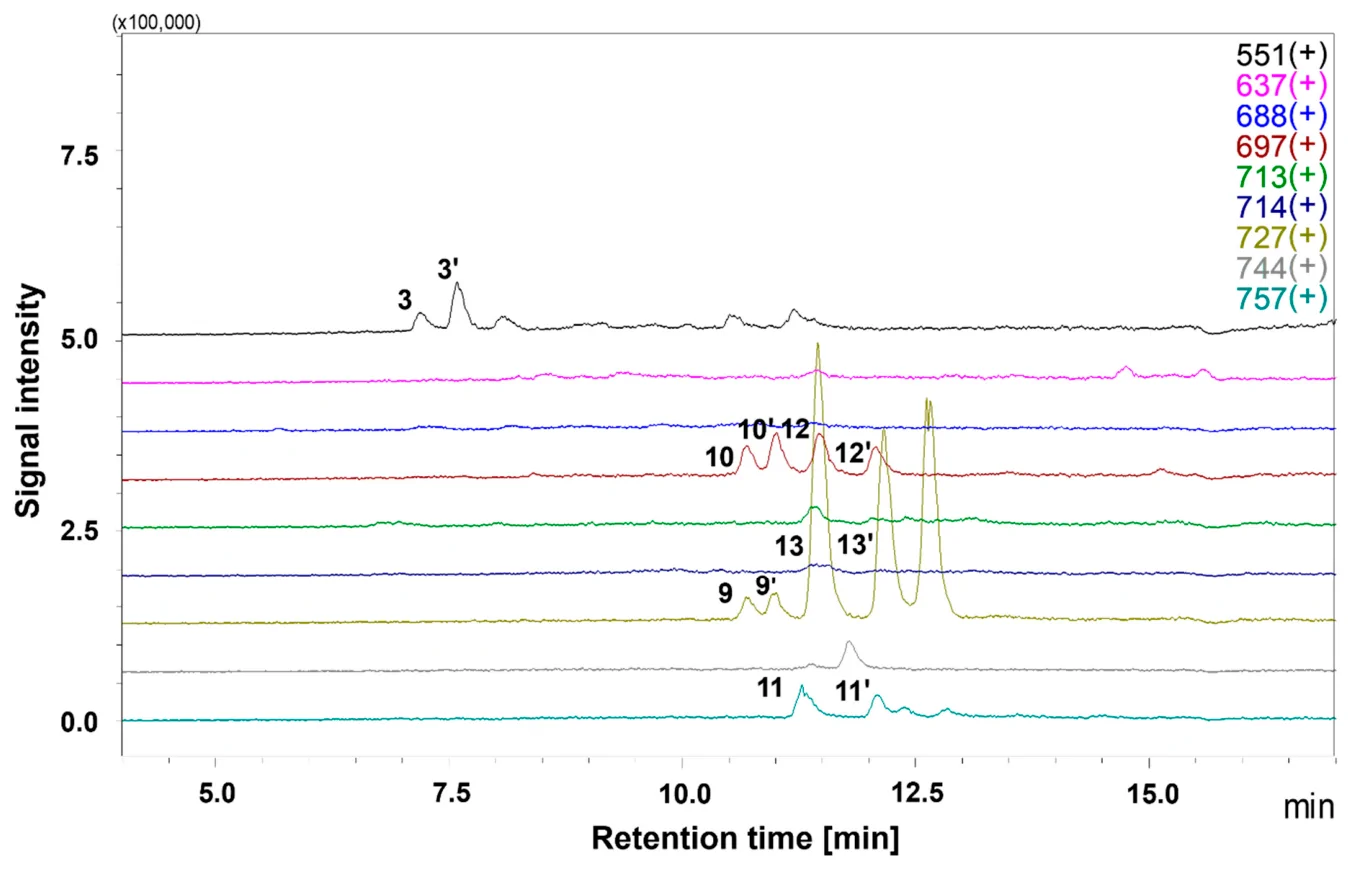
Chromatogram of selected ions monitored in the LC-MS system for GE preparation. Peak numbers correspond to the compounds listed in Table 1.

The total betacyanin content of the purified *G. globosa* extract (GE) was lower than that of *B. alba* f. *rubra*, while the pigment composition differed substantially (Figure 5). Unlike BE_1_, GE contained relatively low amounts of non-acylated gomphrenin **3** (2.2 ± 0.1%) and isogomphrenin **3′** (5.6 ± 0.5%), whereas acylated derivatives collectively predominated in the betacyanin profile. The major betacyanin was basellin **13** (36.4 ± 1.1%) followed by its isoform, isobasellin **13′** (25.4 ± 0.9%), globosin **12** (5.6 ± 0.4%), and sinapoyl derivative gandolin **11** (4.3 ± 0.4%) (Table 2). This betacyanin profile agrees with previous reports indicating that *G. globosa* accumulates predominantly acylated gomphrenin derivatives [21], whereas *B. alba* fruits are characterized by the predominance of non-acylated gomphrenin [22].

The chromatographic profiles also reflect the influence of ion-exchange chemistry on pigment isolation (Figure 1, Figure 2, Figure 3, Figure 4 and Figure 5). Weak anion-exchange purification (BE_1_) retained the complex profile of betacyanins, while the subsequent preparative HPLC enabled fractionation according to polarity, yielding fractions enriched either in the parent glucosides (BE_2_) or in hydroxycinnamoyl derivatives (BE_3_). The applied purification strategy provided an efficient approach for obtaining betacyanin preparations differing markedly in chemical composition for further studies on the physicochemical or biological properties.

### 3.2. Bioactivity

**Figure 6:**
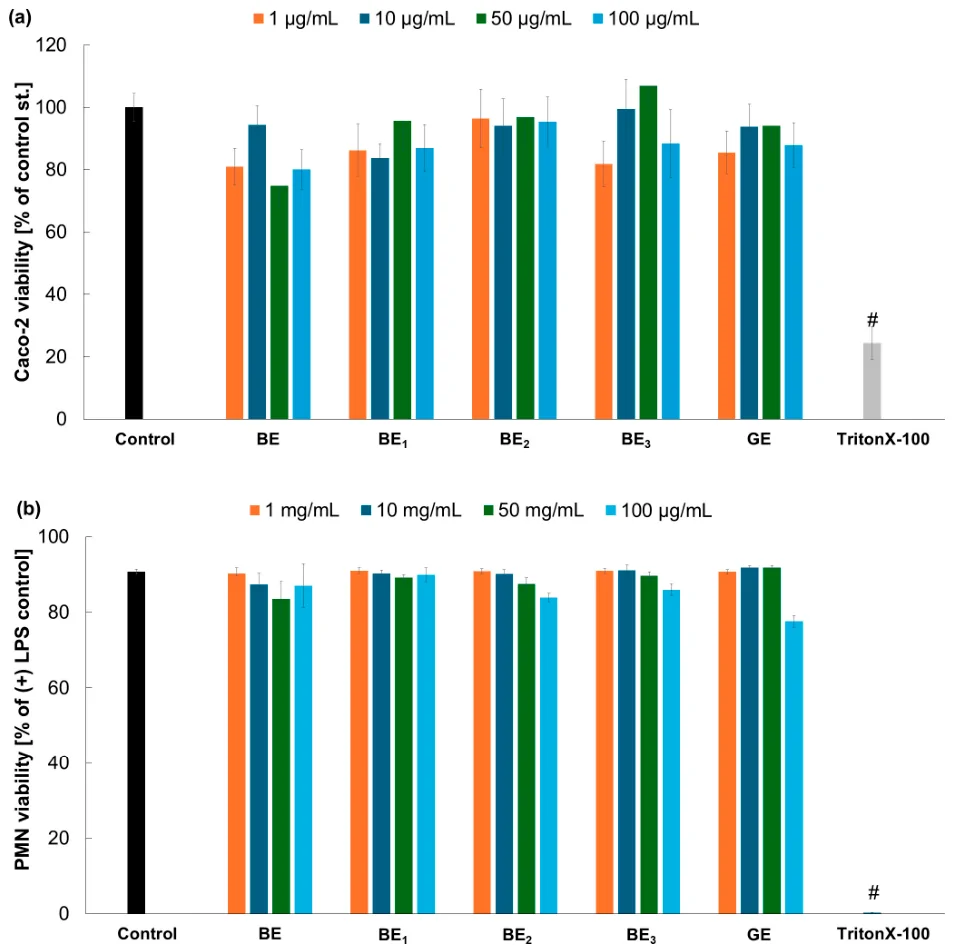
Viability of Caco-2 (**a**) and PMN (**b**) treated with extracts. # statistically significant differences with *p* < 0.001 vs. all treatments.

**Figure 7:**
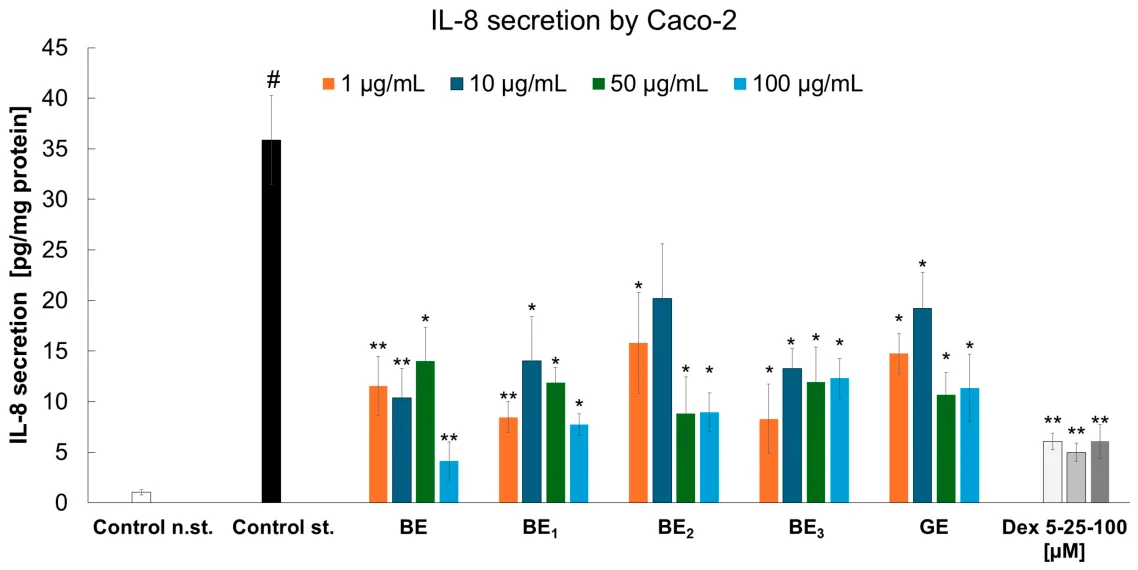
Effect on the secretion of IL-8 in Caco-2. Dex—dexamethasone; # *p* < 0.001 vs. control n.st.; * *p* < 0.05 vs. control st., ** *p* < 0.001 vs. control st.

**Figure 8:**
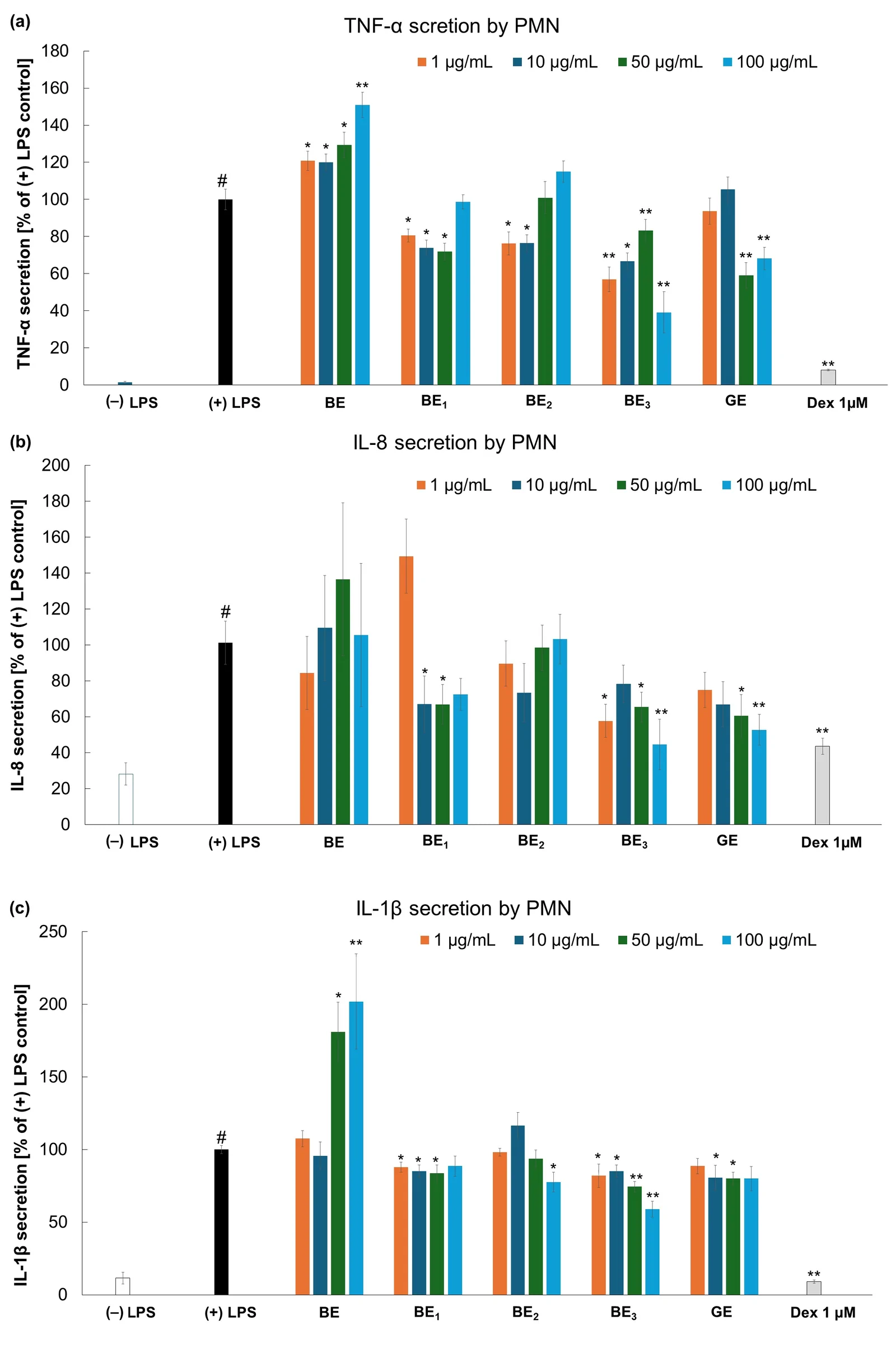
Effect on the secretion of TNF-*α* (**a**), IL-8 (**b**), IL-1*β* (**c**) in PMN. Dex—dexamethasone; # *p* < 0.001 vs. (−) LPS; * *p* < 0.05 vs. (+) LPS, ** *p* < 0.001 vs. (+) LPS.

None of the tested samples affected the viability of Caco-2 or PMN cells over the investigated concentration range (Figure 6), indicating that the observed effects on cytokine secretion (Figure 7 and Figure 8) and ROS generation (Figure 7) were not attributable to cytotoxicity. In particular, the Caco-2 and PMN cells maintained viability above 85% and 90%, respectively, after betacyanin-enriched fraction treatment. Differences in biological responses may therefore reflect variations in betacyanin profiles as well as differences in the overall composition and abundance of other bioactive constituents of the tested preparations.

Purification of the crude *B. alba* extract BE significantly altered its biological properties, as evidenced by a marked reduction in the secretion of cytokines/chemokines, particularly TNF-*α* and IL-8, and IL-1*β* to a lesser extent (Figure 8). This enhanced anti-cytokine activity may be attributed to both the removal of potentially interfering matrix components and the enrichment of bioactive compounds in BE_1_.

**Figure 9:**
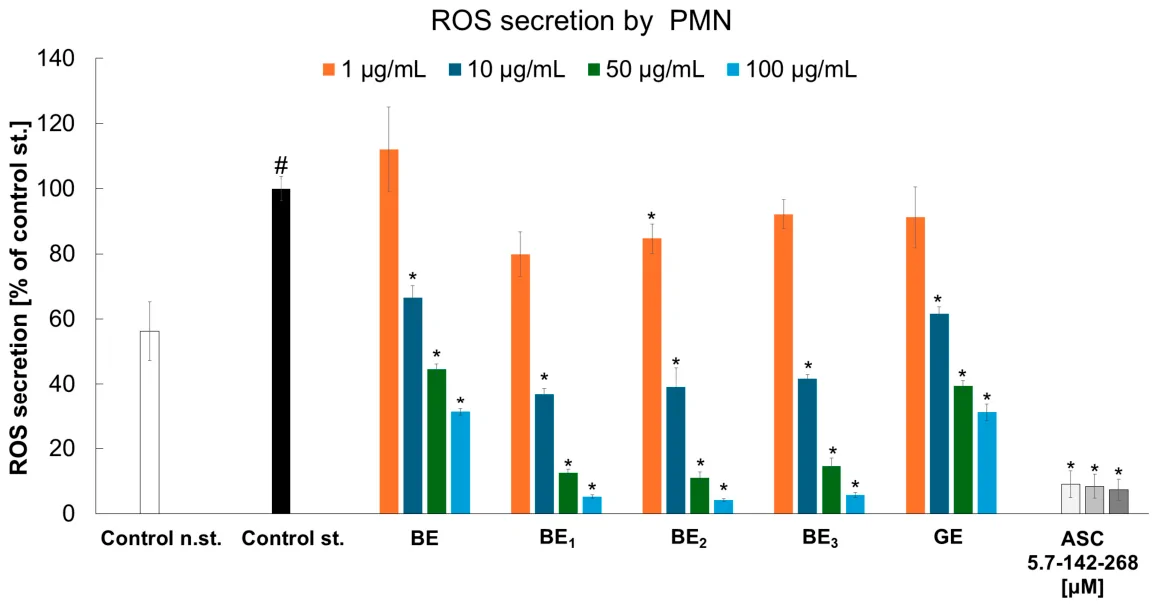
f-MLP stimulated ROS production by PMN. ASC—ascorbic acid; # *p* < 0.001 vs. (−) f-MLP; * *p* < 0.05 vs. (+) f-MLP.

Comparison of the *B. alba* preparations further suggests that differences in betacyanin composition may contribute to their distinct biological effects. In particular, BE_3_, enriched in acylated betacyanins, generally induced a more pronounced reduction in TNF-*α*, IL-8, and IL-1*β* secretion than the gomphrenin-enriched preparation BE_2_ (Figure 8). The effect was statistically significant compared with the LPS-stimulated positive control at most of the tested concentrations. TNF-*α*, IL-8 (CXCL8), and IL-1*β* secretion in BE_3_ (100 μg/mL) was 39.17%, 44.61%, and 58.93% compared to control (*p* < 0.001), whereas BE_2_ (100 μg/mL) exhibited only limited activity (IL-1*β*: *p* < 0.05). In contrast, both preparations effectively suppressed ROS generation by activated PMNs (Figure 9). These findings indicate that the biological activity of the preparations may depend not only on the total betacyanin content but also on the relative abundance and structural characteristics of individual betacyanins. In addition, the anti-cytokine/chemokine effect may not be related only to the antioxidant activity of BE_2_ and BE_3_.

Despite its considerably lower total betacyanin content, GE also exhibited anti-cytokine/chemokine activity (Figure 8). Because this preparation was obtained from a different botanical source and was naturally enriched in acylated betacyanins, direct comparison with the *B. alba*-derived preparations should be interpreted with caution. Moreover, extracts of *G. globosa* flowers may contain a range of other bioactive phenolic constituents, including flavonoids such as quercetin, kaempferol and isorhamnetin derivatives, as well as hydroxycinnamic acids, which could contribute to the observed biological effects [23]. Nevertheless, the similar biological responses observed for GE and BE_3_ support the possibility that hydroxycinnamoyl-substituted betacyanins contribute at least in part, to the modulation of inflammatory responses. It is worth noting that the potency of GE antioxidant activity is much weaker than BE_2_ or BE_3_. Due to the lower gomphrenin **3** and isogomphrenin **3′** content in GE, compared to BE_2_ and BE_3_, it might be suggested that these phytochemicals are mainly responsible for the antioxidant activity in the tested fractions.

All tested preparations significantly reduced IL-8/CXCL8 secretion in the Caco-2 model. All fractions at the highest concentrations decreased the chemokine secretion by more than 50%. CXCL8 is the most potent human neutrophil-attracting chemokine and plays crucial roles in the response to infection and tissue injury. The reduction in IL-8/CXCL8 secretion by gastrointestinal-related cells might lead to limitation of leukocyte recruitment to the site of inflammation and support termination of the inflammatory state.

## 4. Discussion

The LC-DAD-ESI-MS analysis demonstrated that the purification procedures resulted in preparations with markedly different betacyanin profiles, both in terms of total pigment concentration and, more importantly, in the relative abundance and structural diversity of individual betacyanins (Table 1). This chemical differentiation provides an important basis for interpreting the biological effects observed in the cellular models. Rather than representing preparations differing only in the amount of betacyanins, BE_1_, BE_2_, BE_3_, and GE constituted chemically distinct pigment mixtures, characterized by different proportions of non-acylated, malonylated, and hydroxycinnamoyl-substituted betacyanins (Table 2). Consequently, the biological differences observed among these preparations should be considered in the context of both their quantitative pigment content and their qualitative composition.

The crude freeze-dried *B. alba* fruit extract (BE) contained 52 ± 4 mg of total betacyanins per g dry weight, whereas purification substantially increased the pigment concentration in all *B. alba*-derived preparations (Table 2). Thus, the purification procedures increased the total betacyanin concentration approximately 4.2-, 5.5-, and 3.4-fold relative to the crude extract, respectively. This enrichment confirms the effectiveness of the chromatographic procedures but also demonstrates that purification resulted in preparations with substantially different pigment concentrations. A comparable enrichment effect was reported by Xu et al. [24], who observed an increase in betacyanin content from 9.28 ± 0.04 mg/g in a crude red dragon fruit peel extract to 229.45 ± 10.12 mg/g after solid-phase extraction (SPE), corresponding to an approximately 25-fold increase. These findings support the effectiveness of purification in concentrating betacyanins, while highlighting that the magnitude of enrichment may vary considerably depending on the plant source and purification strategy [24].

The chemical composition of BE_1_ indicated that the initial purification step retained a broad spectrum of *B. alba*-derived betacyanins. Although BE_1_ contained numerous compounds, including non-acylated, malonylated, and hydroxycinnamoyl-substituted derivatives, its profile was strongly dominated by gomphrenin 3 (betanidin 6-*O*-*β*-glucoside, Table 2), which accounted for 69.7 ± 1.1% of the detected betacyanins. Isogomphrenin (compound 3′, Table 2) represented a further 13.1 ± 1.5%. Thus, approximately 83% of the betacyanin profile of BE_1_ consisted of these two non-acylated glucosides. The remaining fraction comprised a diverse set of minor pigments, including malonylated and hydroxycinnamoyl derivatives. BE_1_ can therefore be considered a broadly enriched *B*. *alba* betacyanin preparation dominated by non-acylated gomphrenin-type pigments rather than a chemically homogeneous fraction. The predominance of gomphrenin and isogomphrenin in BE_1_ is consistent with previous reports on the betacyanin composition of *B. alba* fruits. Sutor-Świeży et al. [22] reported gomphrenin and isogomphrenin as major pigments in *B. alba* fruits, accounting for 39.7% and 13.7% of the total betacyanin content, respectively, while also identifying a diverse range of malonylated and hydroxycinnamoyl-acylated gomphrenin derivatives. Similarly, Lin et al. [25] identified gomphrenin I as the principal purple pigment of *B. alba* fruits, confirming the characteristic predominance of gomphrenin-type betacyanins in this species [22,25].

In contrast, the preparative HPLC procedure used to obtain BE_2_ resulted in selective enrichment of the two non-acylated glucosides. Gomphrenin and isogomphrenin represented 51.9 ± 4.8% and 47.9 ± 4.8% of the detected betacyanins, respectively, while only trace amounts of malonylated gomphrenin remained. Consequently, more than 99% of the quantified betacyanin profile of BE_2_ consisted of these two structural isomers. This is an important feature of BE_2_ because it makes this preparation considerably more chemically defined than BE_1_ and BE_3_. At the same time, the approximately 1:1 ratio of gomphrenin to isogomphrenin means that the biological effect of BE_2_ cannot be assigned specifically to gomphrenin itself. Any activity of this fraction may reflect the combined effects of both isomers and their possible interactions. The selective enrichment of gomphrenin-type pigments by preparative HPLC is consistent with previous studies demonstrating that chromatographic fractionation can yield chemically defined *B. alba* betacyanin preparations enriched in specific gomphrenin derivatives. Sutor-Świeży et al. [18]) isolated highly purified gomphrenin and several acylated gomphrenin derivatives from *B. alba* extracts and evaluated their bioactivities individually, highlighting the importance of distinguishing the effects of individual pigments from those of complex pigment mixtures. This is particularly relevant for BE_2_, where the nearly equimolar presence of gomphrenin and isogomphrenin prevents the observed biological activity from being unequivocally attributed to gomphrenin alone [18].

BE_3_ exhibited a fundamentally different chemical profile. In contrast to BE_2_, it was strongly enriched in acylated betacyanins, particularly hydroxycinnamoyl derivatives (Table 2). Globosin (*6′*-*O*-*E*-4-coumaroyl-gomphrenin, Table 2) represented 48.3 ± 0.5% of the detected betacyanins, while isoglobosin (compound 12′, Table 2) accounted for 19.8 ± 0.4%. Basellin (*6′*-*O*-*E*-feruloyl-gomphrenin, compound 13 Table 1) and isobasellin (compound 13′) represented 12.8 ± 0.1% and 4.4 ± 0.1%, respectively. Additional acylated derivatives, including malabarin/isomalabarin and other conjugates, were also present, whereas gomphrenin accounted for only 4.6 ± 0.3 mg/g DW. Thus, BE_3_ represents a chemically distinct preparation in which hydroxycinnamoyl-substituted betacyanins predominate over the parent non-acylated glucosides. The lower total betacyanin concentration of BE_3_ compared with BE_2_ is therefore accompanied by a major qualitative shift in pigment composition.

This difference between BE_2_ and BE_3_ is particularly relevant to the biological results. BE_2_ contained the highest total concentration of betacyanins (312 ± 83 mg/g DW), whereas BE_3_ contained significantly less (188 ± 12 mg/g DW). Nevertheless, BE_3_ generally produced a stronger reduction in TNF-α, IL-8, and IL-1β secretion than BE_2_ (Figure 8). The preparations were applied to cells at identical mass concentrations; thus, observations cannot be explained simply by the total amount of betacyanin delivered to the cells. BE_2_ would, on a mass basis, provide considerably more total betacyanin than BE_3_, yet BE_3_ possessed a more complex betacyanin profile and displayed greater activity against several inflammatory endpoints. This finding therefore suggests that differences in the qualitative composition of the preparations, including their betacyanin profile, may be more important than total betacyanin concentration for some biological response. This interpretation is consistent with previous studies indicating that the biological activity of betalain-containing preparations cannot be explained solely by their total pigment content. Smeriglio et al. (2021) [26] demonstrated that betalain-rich extracts could exert stronger anti-inflammatory effects than their major isolated betalains when compared at equivalent concentrations, suggesting that interactions among different constituents may contribute to the overall biological response. Similarly, Fernando et al. [27] showed that individual betalain pigments differed in their effects on inflammatory and oxidative-stress markers, indicating that the biological response depends on the structural characteristics of the pigments. Furthermore, Martinez et al. [28] demonstrated that a betalain-rich preparation from *Beta vulgaris* significantly reduced TNF-*α* and IL-1*β* production in an experimental model of inflammation, confirming that betalain-rich preparations can directly modulate key inflammatory mediators. Taken together, these findings support the hypothesis that the qualitative composition of BE_2_ and BE_3_ may contribute to their different anti-inflammatory effects. Thus, the higher total betacyanin concentration of BE_2_ does not necessarily translate into stronger biological activity, and the relative abundance and structural diversity of individual betacyanins should be considered when interpreting the biological effects of *B. alba*-derived preparations [26,27,28].

Although BE_1_ contained a high total betacyanin concentration (235 ± 57 mg/g DW) and was dominated by gomphrenin, it also retained a significantly greater structural diversity than BE_2_. Its biological properties were therefore likely to reflect the combined effects of major non-acylated pigments and a range of minor derivatives. The fact that purification from BE to BE_1_ significantly altered the inflammatory response indicates that the biological properties of the crude extract cannot be predicted solely from its total pigment content. The crude extract contained only 52 ± 4 mg/g DW total betacyanins, but it also contained a complex matrix of non-pigment constituents that were largely removed or altered during purification. The increased activity of purified preparations may therefore result from pigment enrichment, removal of inhibitory or competing matrix components, improved accessibility of pigments, or changes in pigment stability. Similar observations have been reported for other betalain-containing systems, in which the surrounding matrix influenced pigment stability, accessibility, and biological behavior. Herbach et al. [29] demonstrated that the juice matrix substantially affected the stability and structural transformations of betacyanins, including betanin isomerization and phyllocactin deacylation, highlighting the importance of non-pigment components in determining pigment reactivity. More recently, Roman-Maldonado et al. [30] showed that the composition and complexity of the food matrix significantly affected betalain stability and bioaccessibility, demonstrating that a higher initial pigment concentration does not necessarily translate into greater availability of the compounds at their biological site of action. In addition, Smeriglio et al. [26] reported that betalain-rich extracts could exhibit stronger anti-inflammatory effects than individual major betalains, suggesting that interactions among constituents may contribute to the overall biological response. Therefore, the increased activity observed after purification of BE may reflect not only the approximately fourfold enrichment of betacyanins but also changes in the surrounding chemical environment of the pigments. Removal of non-pigment constituents may have reduced compounds capable of promoting pigment degradation or interfering with their activity, while simultaneously increasing pigment accessibility and stability. However, because these matrix components were not individually characterized in the present study, their contribution to the observed difference between BE and BE_1_ should be regarded as a probable explanation rather than a demonstrated mechanism [26,29,30].

The comparison of BE_2_ and BE_3_ is particularly informative because these fractions represent relatively distinct chemical profiles. BE_2_ consisted almost exclusively of the non-acylated gomphrenin/isogomphrenin pair, whereas BE_3_ was dominated by acylated derivatives, principally globosin, isoglobosin, basellin, and isobasellin. The stronger inhibition of TNF-*α*, IL-8, and IL-1*β* secretion by BE_3_ therefore raises the possibility that hydroxycinnamoyl substitution contributes to the modulation of inflammatory signaling. This interpretation is supported by previous work on *B. alba* gomphrenins, in which the acylated pigments globosin and malabarin exhibited distinct anti-inflammatory activities and inhibited the secretion of IL-1*β* and TNF-*α* in LPS-activated human macrophages [18]. The present results and previous evidence suggest that the higher anti-inflammatory activity of BE_3_ may be related not simply to the total amount of betacyanins, but to the structural characteristics of its predominant acylated gomphrenins. However, because BE_3_ contains several acylated pigments and their isoforms simultaneously, the present data do not allow the activity to be attributed to a specific compound or to hydroxycinnamoylation alone. Acylation, particularly with hydroxycinnamoyl residues, can substantially modify the physicochemical properties of betalains and may consequently influence their stability and biological behavior. In *B. alba*, acylated gomphrenins such as globosin and malabarin have been shown to exhibit greater stability than non-acylated gomphrenin, while also displaying distinct antioxidant and anti-inflammatory activities, indicating that hydroxycinnamoyl substitution can affect both pigment persistence and biological function [18]. This enhanced stability has been attributed, at least in part, to structural effects associated with acylation and intramolecular interactions that may protect the betalain chromophore from degradation [11]. More broadly, the bioavailability and biological effects of betalains depend on their absorption, metabolism, and interactions with biological systems, although these processes have been studied much more extensively for non-acylated pigments than for hydroxycinnamoyl-substituted betacyanins. Therefore, the possibility that acylation influences membrane interactions, cellular uptake, metabolism, or the modulation of specific signaling pathways cannot be excluded, but these mechanisms require direct experimental verification for individual acylated *B. alba* betacyanins.

BE_2_ and BE_3_ differ in several structural types of acyl substituent, the relative abundance of individual acylated derivatives, and the total pigment concentration. Consequently, differences in minor pigments, phenolic constituents, degradation products, or other matrix components may account for the observed effects. Therefore, the relationship between acylation and biological activity should be regarded as an association requiring further experimental verification. Additionally, both preparations effectively reduced ROS generation in activated neutrophils. This difference is potentially important because it suggests that modulation of inflammatory mediator release and suppression of oxidative burst may be partially separable biological activities.

GE contained the lowest total betacyanin concentration among all analyzed preparations (Table 2). Nevertheless, it exhibited measurable biological activity in the cellular models (Figure 8). Its pigment profile was fundamentally different from that of the *B. alba*-derived preparations. Non-acylated gomphrenin and isogomphrenin accounted for only 2.2 ± 0.1% and 5.6 ± 0.5%, respectively, whereas acylated pigments predominated (Table 2). Particularly interesting was the high abundance of feruloylated derivatives: basellin and isobasellin represented 36.4 ± 1.1% and 25.4 ± 0.9%, respectively, together accounting for more than 60% of the detected betacyanin profile. Additional acylated pigments included globosin/isoglobosin and sinapoylated derivatives such as gandolin and isogandolin (Table 2). The predominance of acylated gomphrenin derivatives is consistent with previous studies of *G. globosa*, in which feruloylated and coumaroylated gomphrenins were identified as characteristic pigments of the species [31]. Moreover, a comparative survey of Amaranthaceae species reported one of the highest proportions of acylated betacyanins in *G. globosa*, further supporting the characteristic enrichment of this species in hydroxycinnamoylated gomphrenin derivatives [20]. Importantly, Silva et al. demonstrated that *G. globosa* inflorescence extracts contain both diverse betacyanins and numerous phenolic compounds and exhibit antioxidant and anti-inflammatory activities, including inhibition of nitric oxide production in LPS-stimulated RAW 264.7 macrophages [32]. These findings are particularly relevant to the interpretation of GE because its biological activity may reflect the combined contribution of betacyanins and non-pigment phytochemicals rather than the activity of betacyanins alone. GE originated from a different species and therefore represents a distinct phytochemical matrix, in which phenolic acids, flavonoids, and other secondary metabolites may contribute to the observed cellular response. The relatively low total betacyanin content of GE, together with its measurable biological activity, therefore provides further evidence that total betacyanin concentration alone is insufficient to predict the biological activity of complex plant preparations.

The comparison between BE_3_ and GE is nevertheless informative from a structure-activity perspective. Both preparations were characterized by a high relative abundance of acylated betacyanins, although their profiles were not identical. BE_3_ was dominated by coumaroylated derivatives, globosin and isoglobosin, whereas GE was dominated by feruloylated derivatives, basellin and isobasellin. Previous studies have shown that acylation can influence the biological properties of betacyanins. For example, Spórna-Kucab et al. reported that naturally occurring acylated gomphrenin derivatives from *G. globosa* exhibited stronger antimicrobial activity than non-acylated gomphrenin/isogomphrenin, suggesting that the acyl substituent may contribute to the biological activity of the pigment [33]. This finding does not directly establish an anti-inflammatory effect of hydroxycinnamoylated betacyanins, but it supports the broader concept of a structure-activity relationship associated with acylation. In addition, the chemically distinct coumaroylated and feruloylated gomphrenins identified in *B. alba* fruits represent structurally related but non-identical pigments, providing a basis for considering the nature of the hydroxycinnamoyl substituent as a potential determinant of biological activity [22]. Consistent with this interpretation, different betalains have been shown to exhibit distinct patterns of cellular uptake, redox activity, and modulation of inflammatory markers, indicating that biological responses may depend on the individual pigment structure rather than on betalain concentration alone [34]. Therefore, if BE_3_ and GE exhibited similar biological effects despite their different dominant acyl substituents, this could suggest that activity is associated more broadly with hydroxycinnamoylated betacyanins, whereas differences in their responses would support a contribution of the specific acyl substituent or pigment structure. Additionally, the comparison of BE_2_ and BE_3_ provides a complementary perspective. Both preparations effectively suppressed ROS generation by activated neutrophils despite their markedly different betacyanin profiles (Table 2, Figure 9). This observation suggests that modulation of neutrophil oxidative responses may be a relatively common property of betacyanin-rich preparations and may not require the predominance of either non-acylated or acylated pigments. Previous work demonstrated that purified betanin significantly reduced ROS production in PMA-stimulated human neutrophils, confirming that betalain exposure can modulate neutrophil oxidative metabolism [35]. Moreover, different betalains have been shown to produce distinct effects on both oxidative and inflammatory cellular responses, suggesting that these endpoints may not necessarily be governed by identical structural determinants [34]. Thus, the similar ROS-inhibitory activity of chemically distinct BE_2_ and BE_3_ fractions may indicate that several structurally different betacyanins are capable of modulating cellular redox responses, whereas the stronger differences observed between BE_2_ and BE_3_ in cytokine secretion suggest that inflammatory signaling may be more sensitive to specific pigment structures or to interactions among pigments and other constituents.

Importantly, suppression of cellular ROS generation should not be equated directly with radical-scavenging activity. In activated neutrophils, ROS production is a regulated cellular process involving NADPH oxidase, and reduction in the measured oxidative burst may result from several mechanisms, including direct scavenging of reactive species, interference with NADPH oxidase activation, or modulation of upstream intracellular signaling. Indeed, Zielińska-Przyjemska et al. [35] suggested that the inhibitory effect of betanin on neutrophil chemiluminescence could involve interference with NADPH oxidase activation rather than representing simple chemical scavenging alone. Neutrophil ROS production is also an essential component of antimicrobial host defense. Activation of NADPH oxidase 2 (NOX2) generates superoxide and downstream oxidants that contribute to microbial killing, although excessive or dysregulated ROS production can also promote tissue injury and inflammation [36]. Consequently, the biological significance of the ROS-inhibitory effects observed here depends on the extent and context of the response. Excessive suppression of neutrophil oxidative activity could theoretically compromise antimicrobial defense, and therefore the present findings should be interpreted as modulation of cellular oxidative activity rather than as unequivocally beneficial antioxidant action. Additional mechanistic studies, including assessment of NADPH oxidase activation and downstream redox signaling, would be required to determine the mechanism underlying the observed inhibition. Moreover, ROS can function as signaling molecules and contribute to amplification of NF-κB- and MAPK-dependent inflammatory responses. Consequently, attenuation of intracellular oxidative stress may indirectly reduce activation of redox-sensitive inflammatory pathways and thereby decrease the expression of cytokines and chemokines, including IL8/CXCL8 [37]. The anti-inflammatory role of betalains through MAPK and NF- κB signaling pathways has been indicated recently [14]. Therefore, the reduction in IL-8/CXCL8 secretion observed after treatment with the BE, BE_1_, BE_2_, BE_3,_ and GE preparations may indicate attenuation of one or more upstream inflammatory signaling pathways. Furthermore, IL-8/CXCL8 plays a pivotal role in intestinal inflammatory responses by acting as a potent neutrophil chemoattractant, thereby promoting neutrophil recruitment and activation at sites of epithelial injury or microbial challenge. This may be particularly relevant because activated neutrophils themselves can generate ROS and release additional inflammatory mediators, creating a positive feedback loop between oxidative stress, epithelial signaling, and immune-cell activation. In our study, Caco-2 cells, which are a carcinoma cell line, were used as a model of differentiation from enterocyte-like cells. However, the anti-cancer properties of betalains were recently discussed by Fatima et al. [14]. It was suggested that molecular pathways, other than MAPK, including the PI3K/Akt/mTOR (phosphoinositide 3-kinase/protein kinase B/mammalian target of rapamycin), were also influenced by betalains, which might be connected with anti-cancer activities. The involvement of betalains in these pathways in our model might also be hypothesized. Directly in Caco-2 cells, beetroot extract and betanin increased BCL2-associated agonist of cell death (BAD) and caspase-3, -8, and -9 expression [38]. Another caspase, such as caspase-1, when it is activated, cleaves pro-IL-1*β* into its active form [37]. In our study, the increased secretion of IL-1*β* (*p* < 0.001) was observed for BE, in contrast to BE_1_, BE_2_, and BE_3_. Therefore, further studies, including assessment of caspase-1 and inflammasome activation, would be required to explain the enormous IL-1*β* release, particularly since it is the most potent inducer of IL-8/CXCL8 secretion by Caco-2 [39].

The absence of significant effects on cell viability is an important observation when interpreting the biological results. None of the tested preparations significantly compromised Caco-2 or neutrophil viability over the investigated concentration range. This supports the interpretation that the observed reductions in cytokine secretion and ROS generation were not primarily caused by overt cytotoxicity. Similar observations have been reported for individual betalains in Caco-2 cells, where betanin, vulgaxanthin I, and indicaxanthin maintained cell viability above 90% at the concentrations tested while simultaneously modulating inflammatory and redox-related markers [34]. Nevertheless, maintenance of overall viability does not exclude minor effects on cellular metabolism, membrane function, or signaling pathways, and additional endpoints would be required to establish cellular tolerance more comprehensively.

The present chemical characterization also provides a more appropriate context for interpreting the previously reported biological activity of gomphrenin-rich preparations. Previous studies have demonstrated antioxidant and anti-inflammatory effects of *B. alba* preparations and purified or enriched gomphrenin derivatives, including modulation of cytokine secretion and cellular inflammatory signaling [18]. The present findings are generally consistent with these observations, but they extend them by showing that preparations with substantially different distributions of non-acylated and hydroxycinnamoylated gomphrenins can induce distinct cellular responses. At the same time, studies comparing betalain-rich plant extracts with individual purified pigments indicate that the activity of a complex preparation may also reflect interactions among several constituents rather than the action of a single dominant pigment [26]. Direct comparison with previous studies remains limited by differences in extraction procedures, pigment composition, experimental concentrations, inflammatory stimuli, and cell models. Despite these promising findings, several limitations should be acknowledged. Although the gomphrenin-enriched preparations reduced IL-8/CXCL8 secretion in Caco-2 cells, the underlying molecular mechanisms were not directly investigated. In particular, the involvement of NF-*κ*B, MAPK, and ROS-sensitive signaling pathways remains hypothetical and requires further validation through analyses of NF-*κ*B activation, MAPK phosphorylation, CXCL8 gene expression, and intracellular ROS generation. Moreover, the relative contributions of individual bioactive compounds and their interactions remain to be elucidated. As the study was conducted using an in vitro intestinal epithelial model, the physiological relevance of the findings requires further investigation. It also remains unclear whether the observed effects result from the intrinsic activity of betalains per se or their metabolites formed in the cellular microenvironment. Moreover, in vivo microbiome-mediated transformations within the intestinal microenvironment may generate metabolites with distinct biological activities. Future studies incorporating microbiome transformation models are therefore needed to clarify these mechanisms and establish the contribution of microbial metabolites to the biological activity of betalain-rich preparations. Overall, the findings suggest that their biological activity depends on the interplay between total pigment content, individual betacyanin structures, acylation patterns, and the broader phytochemical matrix, rather than on total betacyanin concentration alone.

## 5. Conclusions

The present study demonstrates that chromatographic purification of *B. alba* and *G. globosa* extracts led to obtaining betacyanin-rich preparations with markedly different quantitative and qualitative pigment profiles. LC-DAD-ESI-MS analysis confirmed efficient enrichment of distinct structural classes of betacyanins, ranging from non-acylated gomphrenin/isogomphrenin-rich preparations to fractions dominated by hydroxycinnamoyl-substituted derivatives. The biological results indicated that the activity of the investigated preparations cannot be explained solely by their total betacyanin content. BE_2_ contained the highest total concentration of betacyanins and was characterized by a betacyanin profile consisting almost exclusively of gomphrenin and isogomphrenin, whereas BE_3_ contained substantially less total pigment but was enriched in acylated betacyanins and showed stronger inhibition of several pro-inflammatory cytokines. Similarly, GE exhibited biological activity despite its considerably lower total betacyanin content and its distinct, predominantly acylated betacyanin profile. These findings indicated that the qualitative composition and structural characteristics of betacyanins may be important determinants of their cellular activity. It should be emphasized, however, that the investigated preparations were not composed exclusively of betacyanins but represented chemically complex plant-derived matrices containing additional constituents that may also have contributed to the observed biological effects. The strong inhibition of ROS generation observed for the *B. alba* preparations further demonstrates their capacity to modulate oxidative responses in activated neutrophils. However, differences in cytokine inhibition between the preparations indicated that modulation of oxidative burst and inflammatory mediator secretion may not be governed by identical mechanisms. The association between acylated-betacyanin enrichment and enhanced modulation of selected inflammatory endpoints is particularly noteworthy and provides a rationale for further investigation of hydroxycinnamoyl-substituted gomphrenins and related pigments. Nevertheless, because the tested fractions remain chemically complex and differ in both pigment composition and botanical origin, the present results do not establish a direct causal relationship between betacyanin acylation and anti-inflammatory activity. Overall, the findings identify *B. alba* and *G. globosa* as promising sources of structurally diverse betalains with potential to modulate inflammatory and oxidative cellular responses. More importantly, the study highlights that, in addition to total betacyanin content, the qualitative composition of betalain-rich preparations should be considered when evaluating their biological properties. Further studies using purified individual betacyanins, equimolar comparisons, mechanistic assays, and physiologically relevant intestinal models are required to establish definitive structure-activity relationships and to determine the translational relevance of these findings.

## Figures and Tables

**Table 1:** Chromatographic, mass spectrometric and spectrophotometric characteristics of betacyanins detected in the studied samples.

No.	Compound	R_t_ [min]	*m/z* [M+H]^+^	λ_max_ [nm]	Occurrence *
1	betanin	5.6	551	535	BE_1_; BE_3_
2	betanidin 6-*O*-*β*-sophoroside (glabranin)	6.3	713	542	BE_1_
2′	isobetanidin 6-*O*-*β*-sophoroside (isoglabranin)	6.6	713	542	BE_1_
3	betanidin 6-*O*-*β*-glucoside (gomphrenin)	7.3	551	539	BE_1_; BE_2_; BE_3_; GE
3′	isobetanidin 6-*O*-*β*-glucoside (isogomphrenin)	7.7	551	539	BE_1_; BE_2_; BE_3_; GE
4	4′-*O*-malonyl-gomphrenin	8.4	637	536	BE_1_; BE_2_
5	C_9_H_8_NO_4_-gomphrenin	8.8	744	543	BE_1_
4′	4′-*O*-malonyl-isogomphrenin	9.4	637	538	BE_1_
6	C_7_H_8_NO_2_-gomphrenin	10.2	688	538	BE_1_
7	6′-*O*-*E*-caffeoyl-gomphrenin (malabarin)	10.4	713	545	BE_1_; BE_3_
6′	C_7_H_8_NO_2_-isogomphrenin	10.5	688	538	BE_1_
8	C_8_H_6_NO_3_-gomphrenin	10.5	714	542	BE_1_; BE_3_
9	*Z*-isomer of basellin	10.7	727	544	GE
10	*Z*-isomer of globosin	10.8	697	544	BE_1_; BE_3_; GE
7′	6′-*O*-*E*-caffeoyl-isogomphrenin (isomalabarin)	10.9	713	545	BE_1_; BE_3_
8′	C_8_H_6_NO_3_-isogomphrenin	10.5	714	542	BE_3_
9′	*Z*-isomer of isobasellin	11.0	727	544	GE
10′	*Z*-isomer of isoglobosin	11.0	697	544	BE_3_; GE
11	6′-*O*-*E*-sinapoyl-gomphrenin (gandolin)	11.3	757	544	BE_3_; GE
12	6′-*O*-*E*-4-coumaroyl-gomphrenin (globosin)	11.5	697	547	BE_1_; BE_3_; GE
13	6′-*O*-*E*-feruloyl-gomphrenin (basellin)	11.5	727	545	BE_1_; BE_3_; GE
11′	6′-*O*-*E*-sinapoyl-isogomphrenin (isogandolin)	12.1	757	544	BE_3_; GE
12′	6′-*O*-*E*-4-coumaroyl-isogomphrenin (isoglobosin)	12.1	697	547	BE_1_; BE_3_; GE
13′	6′-*O*-*E*-feruloyl-isogomphrenin (isobasellin)	12.2	727	545	BE_1_; BE_3_; GE

* Occurrence of monitored betacyanins in the analyzed preparations.

**Table 2:** Normalized composition of detected betacyanins in the studied preparations, expressed as percentages of the total betacyanin profile determined by LC-MS, and total betacyanin contents determined spectrophotometrically.

No.	Compound	Normalized Betacyanin Concentration [%]			
		BE_1_	BE_2_	BE_3_	GE
1	betanin	0.16 ± 0.06		0.11 ± 0.01	
2	betanidin 6-*O*-*β*-sophoroside (glabranin)	0.48 ± 0.12			
2′	isobetanidin 6-*O*-*β*-sophoroside (isoglabranin)	0.33 ± 0.08			
3	betanidin 6-*O*-*β*-glucoside (gomphrenin)	69.7 ± 1.1	51.9 ± 4.8	4.6 ± 0.3	2.2 ± 0.1
3′	isobetanidin 6-*O*-*β*-glucoside (isogomphrenin)	13.1 ± 1.5	47.9 ± 4.8	2.6 ± 0.2	5.6 ± 0.5
4	4′-*O*-malonyl-gomphrenin	1.0 ± 0.1	0.21 ± 0.03		
5	C_9_H_8_NO_4_-gomphrenin	1.1 ± 0.1			
4′	4′-*O*-malonyl-isogomphrenin	0.53 ± 0.04			
6	C_7_H_8_NO_2_-gomphrenin	0.79 ± 0.03			
7	6′-*O*-*E*-caffeoyl-gomphrenin (malabarin)	1.4 ± 0.1		2.6 ± 0.2	
6′	C_7_H_8_NO_2_-isogomphrenin	0.15 ± 0.02			
8	C_8_H_6_NO_3_-gomphrenin	0.74 ± 0.03		1.0 ± 0.1	
9	*Z*-isomer of basellin				2.3 ± 0.3
10	*Z*-isomer of globosin	0.13 ± 0.07		0.5 ± 0.1	3.5 ± 0.3
7′	6′-*O*-*E*-caffeoyl-isogomphrenin (isomalabarin)	0.26 ± 0.01		1.1 ± 0.1	
8′	C_8_H_6_NO_3_-isogomphrenin			0.39 ± 0.04	
9′	*Z*-isomer of isobasellin				2.8 ± 0.4
10′	*Z*-isomer of isoglobosin			0.2 ± 0.1	5.3 ± 0.1
11	6′-*O*-*E*-sinapoyl-gomphrenin (gandolin)			1.2 ± 0.1	4.3 ± 0.4
12	6′-*O*-*E*-4-coumaroyl-gomphrenin (globosin)	7.0 ±0.6		48.3 ± 0.5	5.6 ± 0.4
13	6′-*O*-*E*-feruloyl-gomphrenin (basellin)	1.7 ±0.1		12.8 ± 0.1	36.4 ± 1.1
11′	6′-*O*-*E*-sinapoyl-isogomphrenin (isogandolin)			0.49 ± 0.08	2.8 ± 0.2
12′	6′-*O*-*E*-4-coumaroyl-isogomphrenin (isoglobosin)	1.1 ± 0.2		19.8 ± 0.4	3.9 ± 0.3
13′	6′-*O*-*E*-feruloyl-isogomphrenin (isobasellin)	0.37 ± 0.03		4.4 ± 0.1	25.4 ± 0.9
Total pigment content [mg/1 g DW]		235 ± 57	312 ± 83	188 ± 12	12 ± 1

Values are presented as the mean ± SD (*n* = 3).

## Data Availability

The data are available on request from the corresponding authors.

## Abbreviations

The following abbreviations are used in this manuscript:

Abbreviation	Full name
BE	*Basella alba* f. *rubra* fruit extract
BE_1_	Betacyanin-enriched *Basella alba* f. *rubra* fruit extract
BE_2_	Betanidin 6-*O*-β-glucoside (gomphrenin)-enriched fraction
BE_3_	6′-*O*-trans-4-coumaroyl-gomphrenin (globosin)-enriched fraction
GE	The enriched extract from dried purple flowers of *Gomphrena globosa*
ACD	Acid-citrate-dextrose solution (also referred to as anticoagulant citrate-dextrose solution)
Caco-2 cells	Human colorectal adenocarcinoma cells; an intestinal epithelial cell model.
DMEM	Dulbecco’s Modified Eagle Medium
DMSO	Dimethyl sulfoxide
EDTA	Ethylenediaminetetraacetic acid
FBS	Fetal bovine serum
f-MLP-stimulated PMN	Formyl-methionyl-leucyl-phenylalanine-stimulated polymorphonuclear neutrophils
HBSS	Hanks’ Balanced Salt Solution
IL-1*β*	Interleukin-1 beta
IL-8	Interleukin-8
IL-8/CXCL8	Interleukin-8/C-X-C motif chemokine ligand 8
LC-DAD-ESI-MS	Liquid chromatography–diode-array detection–electrospray ionization mass spectrometry
LPS	Lipopolysaccharide
MAPK	Mitogen-activated protein kinase
MTT	3-(4,5-Dimethylthiazol-2-yl)-2,5-diphenyltetrazolium bromide.
NaHCO_3_	Sodium bicarbonate (sodium hydrogen carbonate)
NF-κB	Nuclear factor kappa B (nuclear factor kappa-light-chain-enhancer of activated B cells)
PBS	Phosphate-buffered saline
PI3K	Phosphoinositide 3-kinase
Akt	Protein kinase B
mTOR	Mechanistic target of rapamycin (historically, mammalian target of rapamycin)
PMA-stimulated human neutrophils	Phorbol 12-myristate 13-acetate-stimulated human neutrophils
ROS	Reactive oxygen species
RPMI	Roswell Park Memorial Institute medium (commonly RPMI 1640)
TNF-α	Tumor necrosis factor alpha
